# Aneurysms of the P2P Segment of Posterior Cerebral Artery: Case Report and Surgical Steps

**DOI:** 10.1155/2014/325414

**Published:** 2014-12-09

**Authors:** Paulo Aguiar, Luana Gatto, Maick Neves, Carlos Martins, Fabio Nakasone, Gustavo Isolan

**Affiliations:** ^1^Department of Neurosurgery, Santa Paula Hospital, Rua Barata Ribeiro 239/12, Bela Vista, 01332-001 São Paulo, SP, Brazil; ^2^Department of Neurosurgery, Cajuru University Hospital, Pontifícia Universidade Católica do Paraná, Rua Sao Jose 300, Cristo Rei, 80050-350 Curitiba, PR, Brazil; ^3^Department of Surgery, Division of Post Graduation, Federal University of Rio Grande do Sul, Avenida Paulo Gama 110, Farroupilhas, 90040-060 Porto Alegre, RS, Brazil

## Abstract

The posterior cerebral artery (PCA) is divided into 4 segments: precommunicating segment (P1), postcommunicating segment (P2), quadrigeminal segment (P3), and calcarine segment (P4). Small aneurysms are more prevalent than large aneurysms in patients with ruptured aneurysms. P2 and P3 aneurysms are usually managed by the subtemporal approach. This is a case report of rupture saccular aneurysm of posterior cerebral artery on P2P segment. The authors show the surgical steps of these rare aneurysms with an illustrative case.

## 1. Introduction

**Figure 1:**
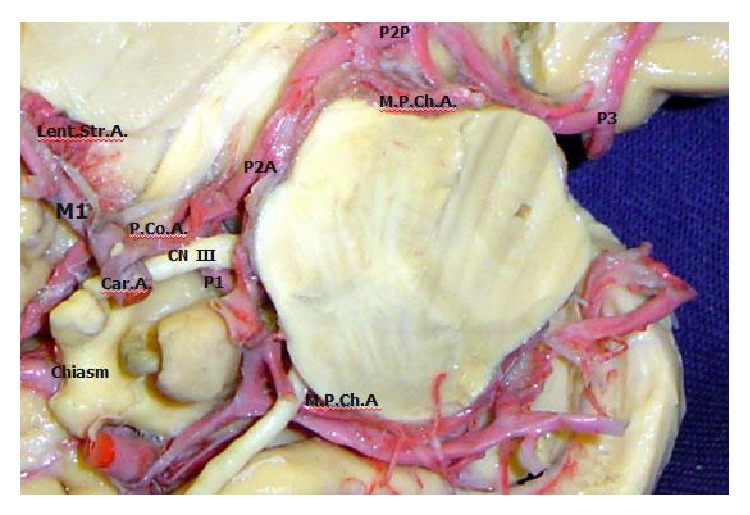
M1: first segment of middle cerebral artery; Lent. Str.A.: lateral lenticulostriates artery from the first segment of middle cerebral artery; Car. A.: internal carotid artery; P.Co.A.: posterior communicating artery; CN III: oculomotor nerve; P1: precommunicating segment of posterior cerebral artery; P2A: crural segment of postcommunicating division of posterior cerebral artery; P2P: ambient segment of postcommunicating division of posterior cerebral artery; M.P.Ch.A.: medial posterior choroid artery; P3: quadrigeminal segment of posterior cerebral artery.

The posterior cerebral artery (PCA) is divided into four segments: P1 (precommunicating segment), P2 (postcommunicating segment), P3 (quadrigeminal segment), and P4 (calcarine segment). The P2 segment is divided into anterior P2A or crural segment and posterior P2P or ambient segment, which together are 25 mm long (Figure 1). The P2 segment of the PCA has in its junction the origin of posterior communicating artery (PComA), with a mean length of 19.9 mm. The P2A begins at the PComA and its way between the peduncle and uncus; the P2P begins at the posterior edge of the cerebral peduncle at the junction of the crural and ambient cistern [1].

The branches of these segments are the peduncular perforating and thalamogeniculate arteries, branches to the ventricles and to the choroid plexus, besides inferior temporal branches (anterior, middle and posterior temporal arteries) [1].

Occurrence of aneurysms in this territory is very rare. Small aneurysms are more prevalent than large aneurysms in patients with ruptured aneurysms. The most common age group is 40–49. The risk of hemorrhage associated with repair of unruptured intracranial aneurysms may depend on the size and location of aneurysm and the probability of aneurysm rupture is proportional to the cube of aneurysm diameter [2].

## 2. Case Report

**Figure 2:**
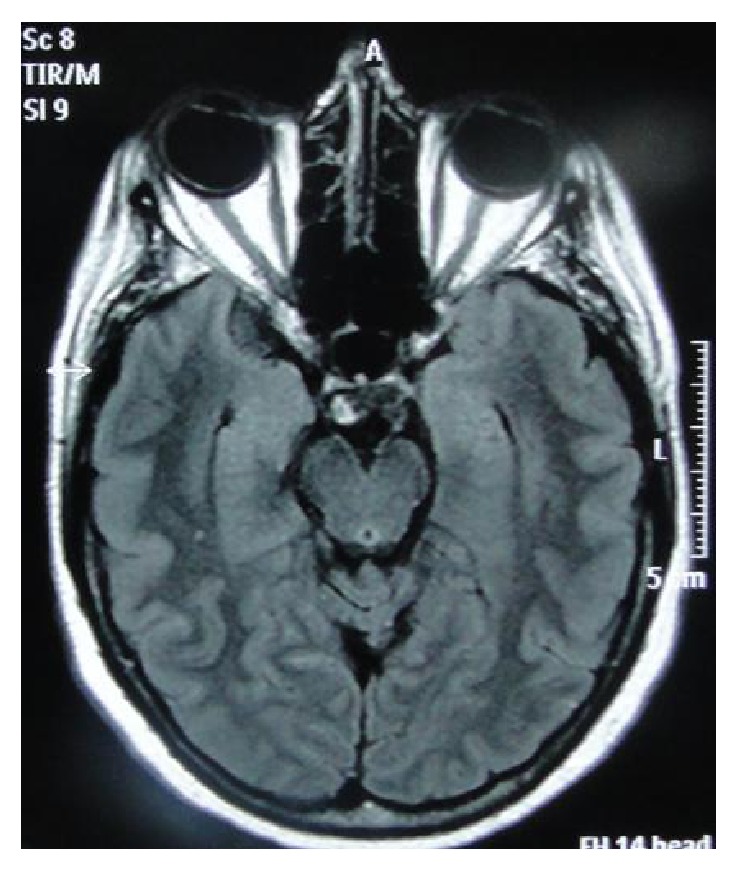
Magnetic resonance without any abnormality.

**Figure 3:**
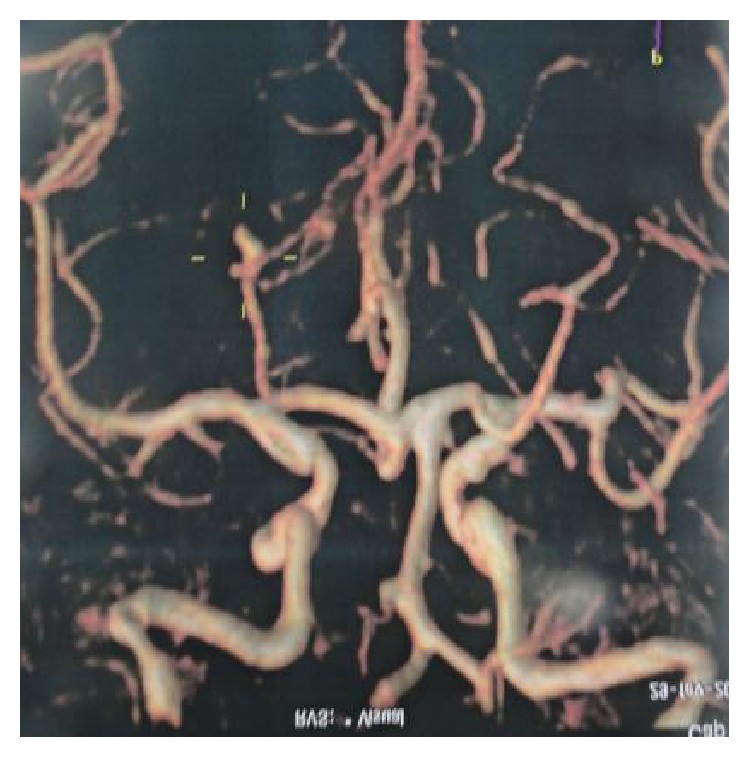
Angiotomography shows a P2P aneurysm of the right side (yellow target).

A sixty-two-year-old female patient was examined due to sudden headache. She is a chronic smoker and was investigated through computed tomography (CT), brain magnetic resonance imaging (Figure 2), angiotomography (Figure 3), and cerebral angiography. The complementary evaluation showed an aneurysmal dilatation on P2P segment in the right side.

**Figure 4:**
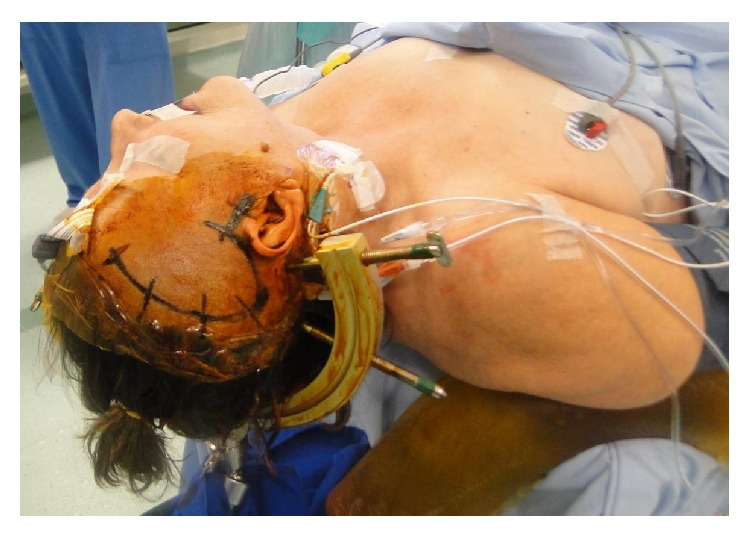
Positioning the patient with Mayfield surgical frame to subtemporal approach.

**Figure 5:**
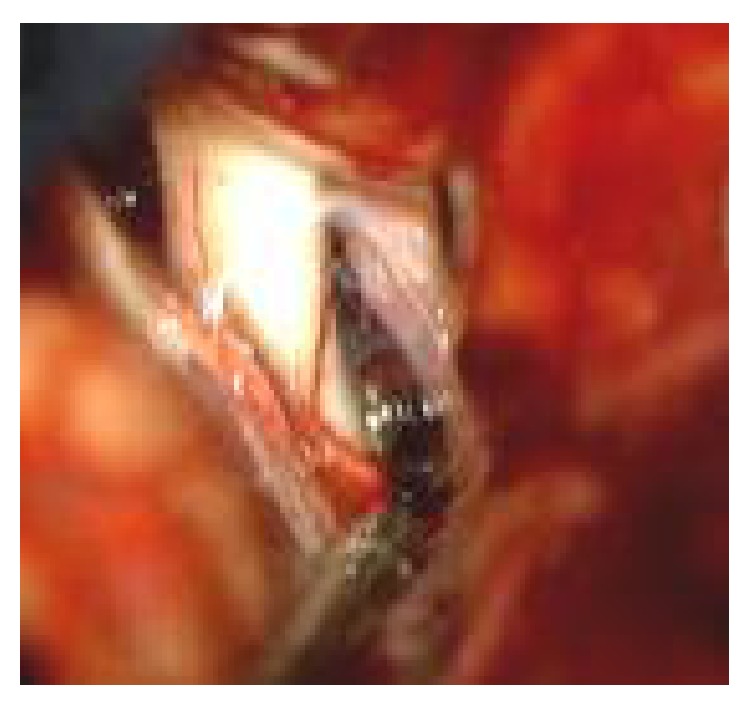
Detaching and mobilizing the anterior temporal lobe open the operative corridor through the carotid-oculomotor triangle.

**Figure 6:**
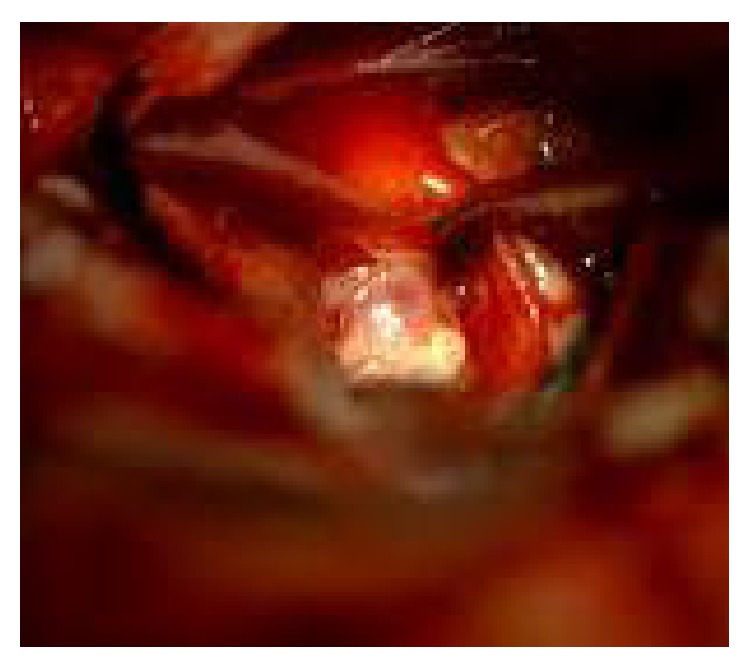
Visualization of the P2P segment aneurysm.

**Figure 7:**
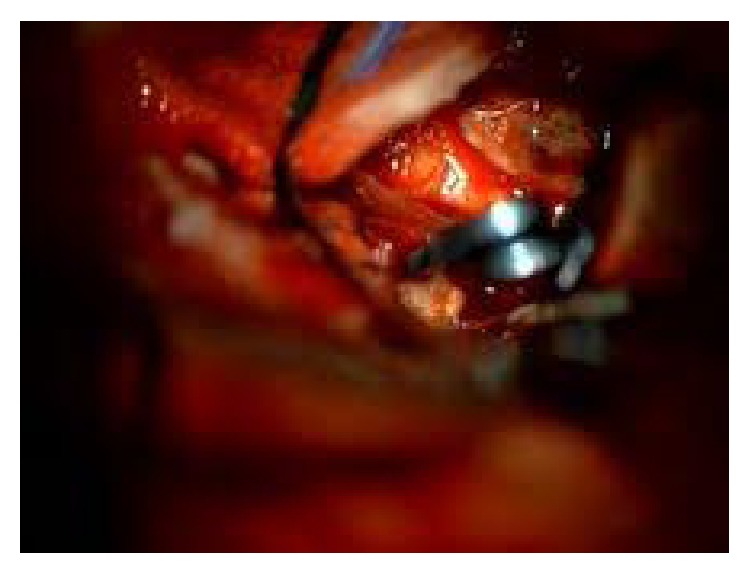
Definitive clipping of the aneurysm.

The patient was positioned with Mayfield surgical frame and submitted to a subtemporal approach (Figure 4). Superficial dissection strategy for P2P aneurysm was done by detaching and mobilizing the anterior temporal lobe opening the operative corridor through the carotid-oculomotor triangle (Figure 5). Splitting the Sylvian fissure the frontal and temporal lobes could be separated. The temporal lobe was mobilized posterolaterally to open the pretemporal corridor. Along the cisternal segment was performed a dissection of the anterior choroidal artery releasing the medial temporal lobe. The deep dissection strategy happens with the identification of the posterior communicating artery, following this artery to the P1-P2 junction. Dissecting the P2 segment was necessary laterally over the oculomotor nerve to the tentorial edge (Figure 6). In the sequence, the P2A segment was followed to the P2P aneurysm. With a dissection of the neck of the aneurysm a definitive Sugita clip (Mizuho, Japan) was placed (Figure 7).

**Figure 8:**
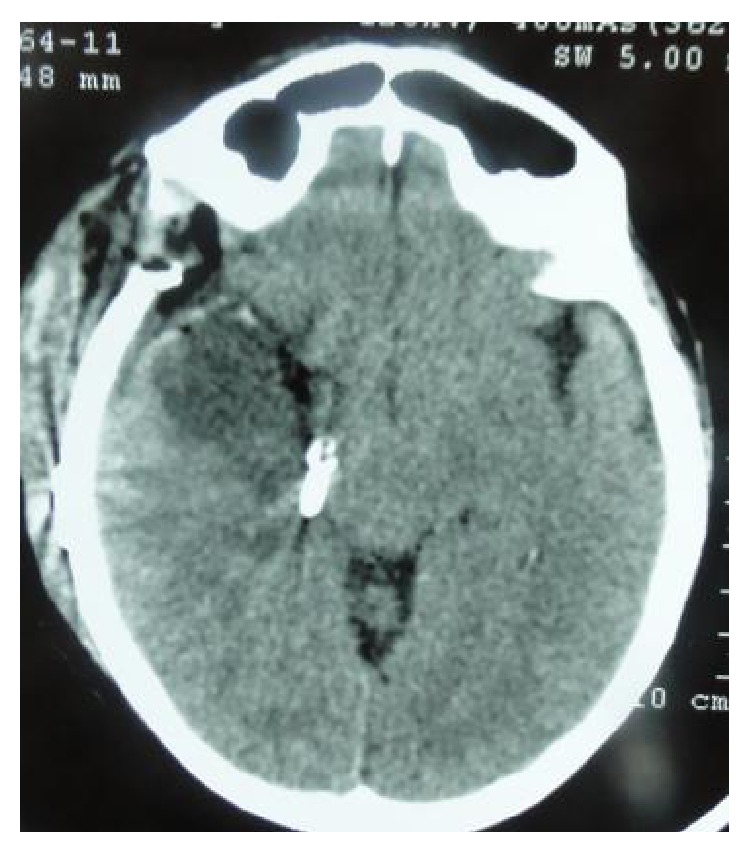
A postoperative CT scan.

**Figure 9:**
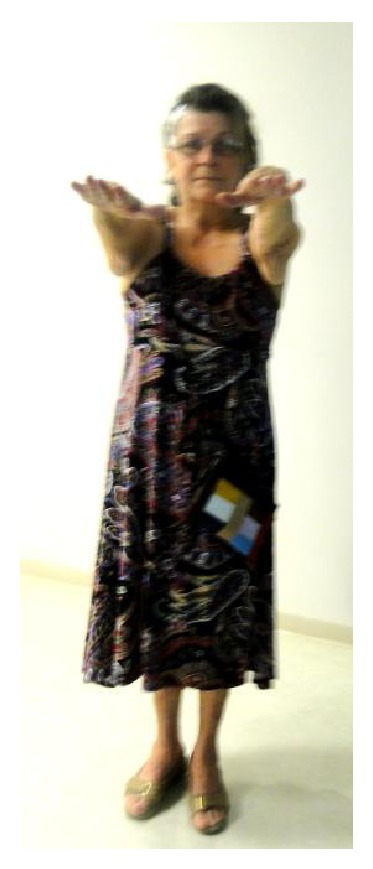
Patient 3 days after the surgery.

After the surgery a CT scan was performed (Figure 8). Patient showed a good postoperative evolution without neurologic deficits (Figure 9).

## 3. Discussion

Patients with aneurysms of the PCA are aged between 38 and 68 years (mean 57.5 years), and regarding gender they are distributed as 1 : 1 female : male [2]. Seventy per cent presented with subarachnoid hemorrhage, 20% are found incidentally, and 10% were associated with cerebral arteriovenous malformations [2].

Helical computed tomographic angiography (CTA) is a relatively noninvasive volumetric imaging technique with higher sensitivity than digital subtraction angiography (DSA) especially for detection of cerebral aneurysms <5 mm in diameter [3]. The posterior cerebral artery aneurysm may be easily identified by CTA before decision making about the treatment [3]. We used for our case the CTA as first diagnostic method before the treatment.

Taking into consideration the deep location of PCA and its close relationship with the brainstem and surrounding vital structures, surgical treatment of aneurysms in this region is complex [4].

In the series of eleven cases of Kitazawa et al., 7 aneurysms were saccular (including one mycotic) and the other four were fusiform [4]. They found that locations of aneurysms were in P1 segment in 2 patients, P1-P2 junction in 2 patients, P2 segment in 6 patients, and P3 in one patient [4]. The predominant location is P1 in 30%, P2 in 30%, P1-P2 junction in 30%, and P3 in 10% [2].

PCA aneurysms are good candidates for direct clipping; however in special cases coil embolization has been indicated as the first choice therapy [2]. In aneurysms placed in curved parent vessels as PCA aneurysms new stents have been developed to reduce the stress against the wall promoted by the intense flow in this territory [5]. PCA aneurysms can be treated with proximal occlusion of the parent artery, excision of aneurysm, or wrapping [4].

Microsurgery for P2 posterior cerebral artery has become a secondary therapy when the neck is unfavorable [6] and the subtemporal approach is a simple kind of treatment in experienced hands [7]. The P2 dissecting aneurysms can be treated with parent vessels occlusion in cases where selective embolization sac with detectable platinum coils or surgical clipping cannot be achieved [8].

Aneurysms of P1 and P1-P2 junction are treated preferably with the pterional approach. P2 and P3 aneurysms are usually handled through the subtemporal approach [9]. Thus, in 11 cases Sakata et al. presented 7 good results, 2 poor results, and one death [9].

Revascularization of the posterior circulation may be performed in order to exclude the aneurysm in the parent vessel with clipping just before the aneurysm occlusion by endovascular balloon [10]. Current indications for revascularization include symptomatic vertebrobasilar ischemia refractory to medical therapy and ischemia caused by parent vessel occlusion as treatment of complex aneurysms [10]. Pretreatment studies may include cerebral blood flow measurements with the assessment of hemodynamic reserve and can affect treatment decisions [10]. The superficial temporal artery, occipital artery, and external carotid artery can be used to increase blood flow to superior cerebellar artery, posterior cerebral artery, posterior inferior cerebellar artery, or anterior inferior cerebellar artery [10].

Severe disability may be found in patients treated through subtemporal approach or temporopolar approach due to temporal lobe contusion [4]. Clipping in P3 aneurysms may develop homonymous hemianopsia due to thrombosis of the parent vessel [2].

## 4. Conclusion

Aneurysms of the P2P segment of PCA are very rare and have a distinct anatomic characteristic. The major treatment for this aneurysm is a direct clipping. The understanding of the complex anatomic of this segment affects their treatment and outcome.
